# Yokukansan Increases 5-HT_1A_ Receptors in the Prefrontal Cortex and Enhances 5-HT_1A_ Receptor Agonist-Induced Behavioral Responses in Socially Isolated Mice

**DOI:** 10.1155/2015/726471

**Published:** 2015-11-23

**Authors:** Toshiyuki Ueki, Kazushige Mizoguchi, Takuji Yamaguchi, Akinori Nishi, Yasushi Ikarashi, Tomohisa Hattori, Yoshio Kase

**Affiliations:** Tsumura Research Laboratories, Kampo Scientific Strategies Division, Tsumura & Co., 3586 Yoshiwara, Ami-machi, Inashiki-gun, Ibaraki 300-1192, Japan

## Abstract

The traditional Japanese medicine yokukansan has an anxiolytic effect, which occurs after repeated administration. In this study, to investigate the underlying mechanisms, we examined the effects of repeated yokukansan administration on serotonin 1A (5-HT_1A_) receptor density and affinity and its expression at both mRNA and protein levels in the prefrontal cortex (PFC) of socially isolated mice. Moreover, we examined the effects of yokukansan on a 5-HT_1A_ receptor-mediated behavioral response. Male mice were subjected to social isolation stress for 6 weeks and simultaneously treated with yokukansan. Thereafter, the density and affinity of 5-HT_1A_ receptors were analyzed by a receptor-binding assay. Levels of 5-HT_1A_ receptor protein and mRNA were also measured. Furthermore, (±)-8-hydroxy-2-(dipropylamino)tetralin hydrobromide (8-OH-DPAT; a 5-HT_1A_ receptor agonist) was injected intraperitoneally, and rearing behavior was examined. Social isolation stress alone did not affect 5-HT_1A_ receptor density or affinity. However, yokukansan significantly increased receptor density and decreased affinity concomitant with unchanged protein and mRNA levels. Yokukansan also enhanced the 8-OH-DPAT-induced decrease in rearing behavior. These results suggest that yokukansan increases 5-HT_1A_ receptors in the PFC of socially isolated mice and enhances their function, which might underlie its anxiolytic effects.

## 1. Introduction

The serotonin (5-hydroxytryptamine, 5-HT) system is widely distributed throughout the brain [1] and is one of the main targets for the pharmacologic treatment of depression, mania, schizophrenia, autism, obsessive-compulsive disorder, and anxiety disorders [2]. Serotonergic signaling is mediated by at least 14 receptor subtypes [3]. Stimulation of 5-HT_1A_ receptors mediates anxiolytic and antiaggressive effects [4, 5]. These 5-HT_1A_ receptors are located both pre- and postsynaptically. Presynaptic 5-HT_1A_ receptors are present on serotonergic neurons as somatodendritic autoreceptors in the dorsal and medial raphe nuclei, whereas postsynaptic 5-HT_1A_ receptors are found at high density in the limbic regions and in the frontal and entorhinal cortices [6, 7]. A serotonergic deficit in the dorsal raphe nuclei and reduced 5-HT_1A_ receptor density in the raphe nuclei and hippocampus were reported in patients with Alzheimer's disease (AD) [8–11]. Lai et al. [12] reported that reduced 5-HT_1A_ receptor- binding in the temporal cortex correlated with aggressive behavior in patients with AD. Furthermore, 5-HT_1A_ receptor agonists have been used successfully for the treatment of anxiety disorders in humans [13].

Yokukansan, a traditional Japanese (Kampo) medicine, is composed of seven dried medicinal herbs. It has been approved by the Ministry of Health, Labour and Welfare of Japan as a treatment for neurosis, insomnia, and night crying and irritability in children. Recent clinical studies reported that yokukansan improves behavioral and psychological symptoms of dementia (BPSD), such as hallucinations, agitation, aggressiveness, and anxiety, in patients with AD, dementia with Lewy bodies, and other forms of senile dementia [14–17]. We previously demonstrated that repeated yokukansan administration (e.g., once a day for 14 days) ameliorated aggressive behaviors in rats injected with the 5-HT neurotoxin para-chloroamphetamine [18] and in mice subjected to isolation stress [19]. Similarly, yokukansan ameliorated anxiety-like behavior in rats subjected to contextual fear conditioning stress [20] or restraint stress [21]. These ameliorative effects were counteracted by the coadministration of the 5-HT_1A_ receptor antagonist WAY-100635, suggesting that the antiaggressive and anxiolytic effects of yokukansan are mediated by 5-HT_1A_ receptor stimulation [18–20]. However, a single administration of yokukansan did not show these effects [19, 21]. These findings suggest that repeated, rather than single, administration of yokukansan is required to express its anxiolytic and antipsychotic effects under certain conditions, implying that some sustained physiological changes related to the 5-HT_1A_ receptor during extended treatment may underlie the psychotropic effects of yokukansan.

To explore the mechanisms underlying the anxiolytic effects of repeated yokukansan administration in isolation-stressed mice, we focused on the 5-HT_1A_ receptors in the prefrontal cortex (PFC), which are involved in emotional behavior [22]. First, we examined the effects of repeated yokukansan administration on 5-HT_1A_ receptor density and affinity and expression at both the mRNA and protein levels in the PFC of mice subjected to social isolation stress. Second, we examined the effects of yokukansan on the 5-HT_1A_ receptor-mediated response, that is, a decrease in rearing behavior induced by the 5-HT_1A_ receptor agonist (±)-8-hydroxy-2-(dipropylamino)tetralin hydrobromide (8-OH-DPAT) in socially isolated mice.

## 2. Materials and Methods

### 2.1. Animals

Male ddY mice, aged 4 weeks, were purchased from Japan SLC, Inc. (Shizuoka, Japan). Mice were housed in groups of five per plastic cage (23 × 31 × 15.5 cm) in a temperature- and relative-humidity-controlled environment (23°C ± 3°C, 55% ± 10%) under a 12 h light-dark schedule and lights on at 7:00 a.m. Mice were fed laboratory food and water* ad libitum* during the habituation and experimental periods.

This study was carried out in accordance with the recommendations in the Guide for the Care and Use of Laboratory Animals of the Japanese Association for Laboratory Animal Science. The protocol was approved by the Committee on the Ethics of Animal Experiments of Tsumura & Co. The experiments in the present study were designed to minimize the number of animals used.

### 2.2. Social Isolation Stress

After habituation for 1 week, the animals were housed individually in transparent plastic cages (11.5 × 31 × 15.5 cm) for 6 weeks. Five other animals were group-housed in a single cage (23 × 31 × 15.5 cm) as controls.

### 2.3. Yokukansan Treatment

Yokukansan is composed of seven dried medicinal herbs:* Atractylodes lancea* Rhizome (4.0 g, rhizome of* Atractylodes lancea* De Candolle), Poria sclerotium (4.0 g, sclerotium of* Poria cocos* Wolf), Cnidium rhizome (3.0 g, rhizome of* Cnidium officinale* Makino), Uncaria Hook (3.0 g, thorn of* Uncaria rhynchophylla* Miquel), Japanese Angelica Root (3.0 g, root of* Angelica acutiloba* Kitagawa), Bupleurum Root (2.0 g, root of* Bupleurum falcatum* Linne), and Glycyrrhiza (1.5 g, root and stolon of* Glycyrrhiza uralensis* Fisher). It was supplied by Tsumura & Co. (Tokyo, Japan) as a dry powdered extract. Each plant used in the preparation of yokukansan was identified by its external morphology and authenticated against known specimens according to the methods of the Japanese Pharmacopoeia and our company's standards. In brief, the active ingredients from the mixture of seven component herbs were extracted with purified hot water at 95°C for 1 h. The extract solution was separated from the insoluble waste and spray-dried to produce the extract powder. The quality was standardized based on the Good Manufacturing Practices defined by the Ministry of Health, Labour and Welfare of Japan.

The powdered yokukansan was incorporated into food pellets at concentrations of 1% and 3% (w/w) by Oriental Yeast Co., Ltd. (Tokyo, Japan). The mice subjected to isolation stress were divided into two groups; the control group was fed standard pellet chow for rodents (MF; Oriental Yeast Co., Ltd.) for 6 weeks during isolation, and the treatment groups were fed MF containing yokukansan (1% or 3%) for 6 weeks during isolation. The weight of food pellets was measured once a week for 6 weeks, and the volume of yokukansan consumed by each mouse housed individually was calculated and averaged: 5.2 ± 0.1 g/day in the isolation stress group; 5.7 ± 0.2 g/day in the 1% yokukansan group; and 5.6 ± 0.3 g/day in the 3% yokukansan group. We confirmed that there was no significant difference in consumption between the three experimental groups. On the day before behavioral testing or brain tissue sampling, the yokukansan-containing chow was replaced with standard chow to eliminate direct effects of yokukansan components on 5-HT_1A_ receptors. For example, geissoschizine methyl ether (GM) is an indole alkaloid component of Uncaria Hook reported to be a partial agonist for 5-HT_1A_ receptors [19]. Group-housed control mice were fed MF only.

### 2.4. Receptor-Binding Assay

After the 6-week period of isolation stress and drug treatment, the animals were killed by decapitation, and the PFC region was quickly dissected from the whole brain on an ice plate, weighed, immediately frozen on dry ice, and stored at −80°C. On the day of biochemical experiments, tissues were thawed and homogenized on ice in 10 : 1 volume/tissue weight of 50 mM Tris-HCl, pH 7.4. The homogenate was centrifuged at 50,000 ×g for 20 min at 4°C. The pellet was rehomogenized and centrifuged again at the same settings. The resultant pellet was resuspended, and aliquots of the suspension were used for the determination of protein concentration by BCA protein assay reagent (Thermo Fisher Scientific, Waltham, MA, USA).

A 5-HT_1A_ receptor-binding assay was performed in duplicate according to a standard method. Briefly, membrane preparations (approximately 40 mg of protein in a final volume of 1 mL) were incubated with [^3^H]8-OH-DPAT (NET 929, specific activity, 5.0 TBq/mmoL; PerkinElmer Life and Analytical Sciences, Shelton, CT, USA) in 50 mM Tris-HCl, pH 7.4, containing 1% dimethyl sulfoxide, at 37°C for 30 min. The concentration of [^3^H]8-OH-DPAT ranged from 0.06 to 0.97 nM. The reaction was terminated by separation of the free and bound radioligand by rapid vacuum filtration through a Whatman GF/B filter. Each filter was washed three times with 3 mL of 50 mM Tris-HCl, pH 7.4. Nonspecific binding was determined by addition of excess (10 *μ*M) unlabeled 5-HT. The trapped radioactivity was counted in 5 mL of Pico-Fluor Plus scintillation fluid (PerkinElmer Life and Analytical Sciences) for 2 min in a liquid scintillation counter (LS-5000; PerkinElmer Life and Analytical Sciences). The number of binding sites was calculated from the radioactivity values. Specific binding was calculated by subtracting nonspecific binding from total binding. The saturation binding data were analyzed by constructing a Scatchard plot, and the maximal number of binding sites (*B*_max_) and equilibrium dissociation constant (*K*_*d*_) were calculated. *B*_max_ is expressed as pmol of [^3^H]8-OH-DPAT bound/mg protein, and *K*_*d*_ is expressed in nM.

### 2.5. Western Immunoblot Analysis

Western immunoblot analysis was performed according to a standard method. Stored PFC tissues were thawed, and homogenate was prepared below 4°C. Briefly, tissue was homogenized in lysis buffer (20 mM Tris-HCl, pH 7.4, 1 mM EDTA, 1 mM EGTA, 0.1% Triton X-100, and 1% protease inhibitor cocktail (Sigma-Aldrich, St. Louis, MO, USA)) in a 2.0-mL tube using an ultrasonic homogenizer (US-150; Nissei, Tokyo, Japan). Homogenate was then incubated at 4°C for 1 h on a rotating device (PVM-2000; LMS, Tokyo, Japan), followed by centrifugation at 22,000 ×g for 30 min to yield a whole cellular fraction containing cytoplasm and plasma membranes but not nuclei (the supernatant). An aliquot of the soluble fraction was used for the determination of protein concentration by DC protein assay reagent (Bio-Rad Laboratories, Hercules, CA, USA). The samples were denatured in lysis buffer containing NuPAGE LDS sample buffer (Thermo Fisher Scientific) and NuPAGE sample reducing agent (Thermo Fisher Scientific) and subjected to gel electrophoresis.

For immunoblot analysis, the proteins (10 *μ*g/lane) were separated by gel electrophoresis on 4–10% NuPAGE Novex Bis-Tris Gels (Thermo Fisher Scientific) and transferred to polyvinylidene difluoride (PVDF) membranes (Hybond-P, GE Healthcare, Buckinghamshire, UK). Nonspecific binding was blocked by incubation with SuperBlock blocking buffer (Thermo Fisher Scientific) at room temperature for 1 h. Blocking was followed by incubation overnight at 4°C in Can Get Signal Solution 1 (Toyobo, Osaka, Japan) with goat anti-human 5-HT_1A_ receptor antibody (sc-1459, 1 : 1,000; Santa Cruz Biotechnology, CA, USA). The immunolabeled blots were then washed and incubated at room temperature for 1 h in Can Get Signal Solution 2 (Toyobo) with horseradish peroxidase- (HRP-) conjugated rabbit anti-goat secondary antibody (A5420, 1 : 160,000; Sigma-Aldrich). The HRP activity was visualized by Pierce Western Blotting Substrate Plus (Thermo Fisher Scientific) using the Typhoon Imaging System (GE Healthcare). After detection of the 5-HT_1A_ receptor proteins, the PVDF membrane was submerged in stripping buffer (100 mM mercaptoethanol, 2% sodium dodecyl sulfate, 62.5 mM Tris-HCl, pH 6.7) and incubated at 60°C for 30 min with occasional agitation. The stripped PVDF membrane was washed, reblocked, and reincubated with goat anti-human actin antibody (sc-1615, 1 : 10,000; Santa Cruz Biotechnology), followed by incubation with HRP-conjugated rabbit anti-goat secondary antibody (A5420, 1 : 160,000; Sigma-Aldrich). The HRP was visualized by the same procedure described above.

Blot images were analyzed by ImageQuant TL software (GE Healthcare) to quantify the protein levels of 5-HT_1A_ receptor and actin. The amount of 5-HT_1A_ receptor protein was normalized to that of actin in the same lane. Data are expressed as change (%) in 5-HT_1A_ receptor/actin relative to group-housed control mice.

### 2.6. Quantitative Real-Time PCR Analysis

After the 6-week period of isolation stress and drug treatment, the animals were killed, and the PFC region was quickly dissected and stored in RNAlater solution (Sigma-Aldrich) at −20°C. On the day of mRNA measurements, total RNA was extracted from PFC tissue using an RNeasy 96 Universal Tissue Kit (Qiagen, Hilden, Germany) according to the manufacturer's specifications. The concentration of extracted total RNA was determined spectrophotometrically at 260 nm. Reverse transcription was performed using a High Capacity cDNA Reverse Transcription Kit (Thermo Fisher Scientific) according to the manufacturer's specifications on a TAK-TP400 Thermal Cycler (Takara Bio Inc., Shiga, Japan). Next, real-time PCR was performed using TaqMan Gene Expression Master Mix (Thermo Fisher Scientific). The PCR was run on an ABI Prism 7900HT sequence detection system with a 384-well format (Thermo Fisher Scientific). The TaqMan gene expression assay probes used were the 5-HT_1A_ receptor (probe ID Mm00434106_s1) and glyceraldehyde-3-phosphate dehydrogenase (GAPDH; probe ID Mm99999915_g1). The real-time PCR was performed in duplicate for each sample. Differences in amplification were determined using the delta-Ct method. GAPDH was used as an endogenous control to normalize expression levels between samples.

### 2.7. 8-OH-DPAT Treatment

After the 6-week period of isolation stress and drug treatment, 8-OH-DPAT (Sigma-Aldrich) was freshly dissolved in saline and injected intraperitoneally at various doses (0.03, 0.1, and 0.3 mg/kg) 15 min before the open field test described below. The doses of 8-OH-DPAT were determined in our preliminary experiments. Control animals were treated with an equal volume of saline (10 mL/kg body weight).

### 2.8. Open Field Test

The open field area was within a gray-colored box (50 × 50 × 40 cm; Neuroscience, Inc., Tokyo, Japan). It was illuminated by a halogen lamp providing the same light intensity as in the animal facility (approximately 40 lx in the center). The animals were initially placed in the center of the open field. The number of rearing behaviors was recorded for 10 min and analyzed by a computer-based tracking system (Limelight 2, Actimetrics, Wilmette, IL, USA). To evaluate motor activity, the total distance traveled was also recorded.

### 2.9. Statistical Analysis

Values are presented as mean ± SEM. Differences in binding parameters and levels of 5-HT_1A_ receptor protein and mRNA were assessed by one-way ANOVA followed by Dunnett's post hoc tests. Differences in rearing and ambulation following injection of 0 (control), 0.03, 0.1, and 0.3 mg/kg 8-OH-DPAT in each experimental group were assessed by the Steel-Dwass test. Differences in rearing and ambulation between the isolation-stressed control and yokukansan cotreated stressed mice, both of which were injected with 0.3 mg/kg 8-OH-DPAT, were also assessed by the Steel-Dwass test. The threshold of significance level for all statistical analyses was *P* < 0.05.

## 3. Results

### 3.1. Effect of Isolation Stress and Yokukansan on 5-HT_1A_ Receptor Density and Affinity

**Figure 1:**
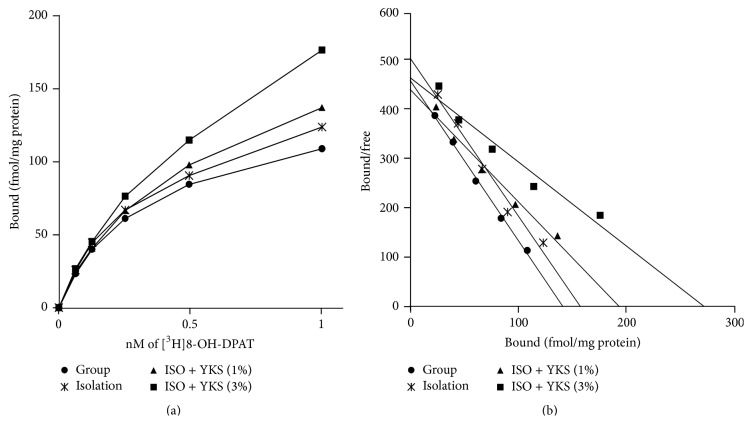
Yokukansan (YKS) cotreatment increased 5-HT_1A_ receptor binding in the prefrontal cortex (PFC) of isolation-stressed mice. Binding of a [^3^H]-labeled 5-HT_1A_ receptor agonist ([^3^H]8-OH-DPAT) in the PFC of group-housed mice (group), mice subjected to isolation stress alone (isolation), or isolation stress with 1% or 3% yokukansan cotreatment (ISO + YKS). (a) Saturation curves and (b) Scatchard plots. Each point is the mean of 10 independent measurements.

The saturation curve and Scatchard plot of [^3^H]8-OH-DPAT binding to PFC membrane preparations are shown in Figure 1. In all experimental groups, total binding gradually increased with rising [^3^H]8-OH-DPAT concentration, and all binding levels were markedly decreased by addition of cold 5-HT, indicating high specific binding. The Scatchard plot analysis indicated that the regression line was linear.

**Figure 2:**
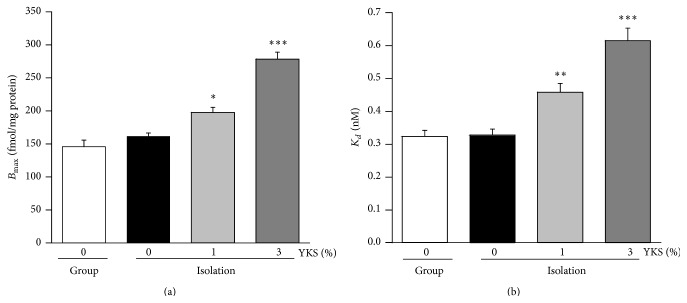
Yokukansan (YKS) cotreatment increased the 5-HT_1A_ receptor density (greater *B*_max_) but reduced receptor affinity (higher *K*_*d*_) in the PFC of isolation-stressed mice. Binding parameters determined by a [^3^H]-labeled 5-HT_1A_ receptor agonist ([^3^H]8-OH-DPAT). (a) *B*_max_ and (b) *K*_*d*_. Each column shows the mean ± SEM (*n* = 10). Asterisks indicate a significant difference: ^*∗*^ *P* < 0.05, ^*∗∗*^ *P* < 0.01, and ^*∗∗∗*^ *P* < 0.001, versus isolation stress alone (0% YKS).

*B*_max_ and *K*_*d*_ values calculated from the Scatchard plot are presented in Figure 2. The *B*_max_ was not changed in isolation-stressed mice compared with group-housed control mice. However, *B*_max_ in isolation-stressed mice was significantly increased by yokukansan cotreatment at concentrations of 1% and 3% (1%: *F*(3,36) = 47.009, *P* < 0.05; 3%: *F*(3,36) = 47.009, *P* < 0.001). Similarly, *K*_*d*_ was not changed by isolation stress alone but was significantly increased by yokukansan cotreatment at concentrations of 1% and 3% (1%: *F*(3,36) = 26.441, *P* < 0.01; 3%: *F*(3,36) = 26.441, *P* < 0.001).

### 3.2. Effect of Isolation Stress and Yokukansan on 5-HT_1A_ Receptor Protein and mRNA Expression

**Figure 3:**
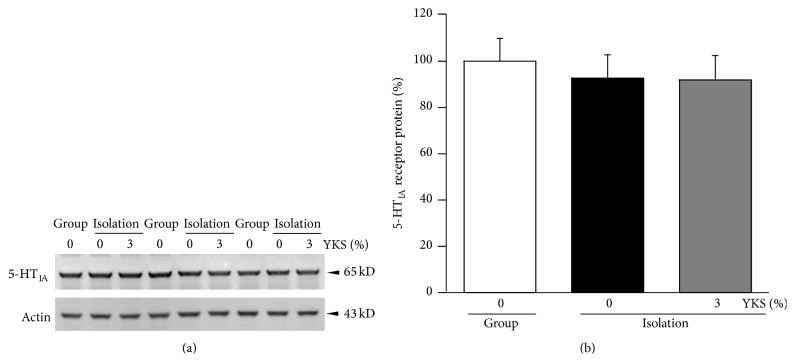
Yokukansan (YKS) cotreatment did not affect expression of 5-HT_1A_ receptor protein in the PFC of isolation-stressed mice. Tissue homogenates for Western blot analysis were prepared from a cell fraction containing cytoplasm and plasma membranes but not nuclei (see Section 2). (a) Representative images of Western blots. Immunoreactive 5-HT_1A_ receptor (65 kD) and actin (43 kD) were detected as single bands. (b) Results of densitometric analysis. Each column is the mean ± SEM (*n* = 10). There were no significant differences among groups.

**Figure 4:**
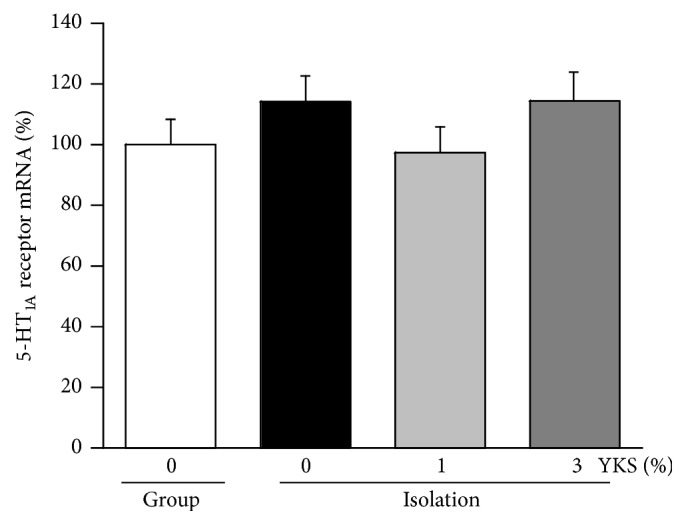
Yokukansan (YKS) cotreatment did not affect expression of 5-HT_1A_ receptor mRNA in the PFC of isolation-stressed mice. Each column is expressed as a percentage of the group-housed control (group, 0% YKS) and mean ± SEM (*n* = 8–10). There were no significant differences among groups.

Figure 3 shows the levels of 5-HT_1A_ receptor protein in tissue preparations containing plasma membrane and cytoplasmic fractions of the PFC. There were no significant differences among groups. Similarly, there were no significant differences in mRNA expression levels among groups (Figure 4).

### 3.3. Effect of Isolation Stress and Yokukansan on 8-OH-DPAT-Induced Response

**Figure 5:**
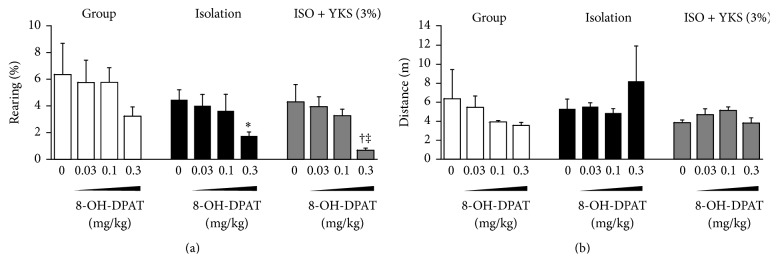
Yokukansan (YKS) cotreatment exacerbated the reduction in rearing induced by injection of a 5-HT_1A_ receptor agonist (8-OH-DPAT) in isolation-stressed mice. (a) Rearing behavior and (b) total distance traveled. Each column is the mean ± SEM (*n* = 9-10). Asterisks and daggers indicate a significant difference: ^*∗*^ *P* < 0.05 versus 0 mg/kg 8-OH-DPAT in mice subjected to isolation stress alone (isolation); ^†^ *P* < 0.05 versus 0 mg/kg 8-OH-DPAT to YKS-treated and isolation-stressed mice [Iso + YKS (3%)]; ^‡^ *P* < 0.05 versus 0.3 mg/kg 8-OH-DPAT-treated isolation-stressed mice.

We examined the effects of isolation stress and yokukansan treatment on rearing behavior and total distance traveled following 8-OH-DPAT injection in the open field. As shown in Figure 5, rearing behavior was not significantly decreased in the group-housed control mice at any dose of 8-OH-DPAT tested (0.03, 0.1, and 0.3 mg/kg) but was significantly reduced by 0.3 mg/kg 8-OH-DPAT in isolation-stressed mice compared with saline-injected isolation-stressed mice (*P* < 0.05). This same 8-OH-DPAT dose significantly decreased rearing in isolation-stressed mice cotreated with 3% yokukansan (*P* < 0.05). Moreover, 3% yokukansan significantly enhanced the effect of 0.3 mg/kg 8-OH-DPAT in isolation-stressed mice, as rearing in response to 0.3 mg/kg 8-OH-DPAT was significantly lower in yokukansan cotreated mice compared with mice subjected to isolation stress alone (*P* < 0.05). In contrast, the total distance traveled following 8-OH-DPAT injection was not affected by isolation stress and yokukansan treatment in 8-OH-DPAT-injected mice at any doses tested.

## 4. Discussion

The current study yielded three major findings: (1) yokukansan increased the density and reduced the affinity of 5-HT_1A_ receptors in the PFC of isolation-stressed mice, while isolation stress alone had no influence; (2) yokukansan cotreatment during isolation stress did not affect the expression of 5-HT_1A_ receptors in the PFC at either the protein or mRNA level; and (3) yokukansan enhanced the 5-HT_1A_ receptor agonist-induced behavioral response, that is, decrease in rearing behavior, in isolation-stressed mice.

The aim of the current study was to explore the functional changes in the brain involved in the anxiolytic effects of repeated yokukansan administration. We found that while isolation stress had no influence on 5-HT_1A_ receptor density in the PFC plasma membrane fraction, yokukansan cotreatment resulted in a significant enhancement in receptor density (Figures 1 and 2). The consumed dose per day of yokukansan, which was fed at 1% in food pellets, corresponded to approximately 1,000 mg/kg/day. This dose was thought to be reasonable to assess the psychotropic effects of yokukansan in mice [19]. The increased density was not due to an increase in total receptor protein or enhanced transcription of the mRNA, as revealed by Western immunoblot analysis of the soluble fraction (protein in the cell membrane and cytoplasm) and PCR analysis (Figures 3 and 4). Rather, we presume that yokukansan may enhance 5-HT_1A_ receptor translocation from the cytoplasm to the plasma membrane. Zhou et al. [23] reported that KIF13A, a molecular motor protein, plays an important role in 5-HT_1A_ receptor membrane translocation. However, neither isolation stress alone nor isolation stress with yokukansan cotreatment affected mRNA expression of KIF13A in the PFC (data not shown). Other possible mechanisms include increased palmitoylation, which promotes retention of 5-HT_1A_ receptors on lipid rafts in cell membrane [24], and reduced SUMOylation, which promotes receptor internalization [25].

In addition to higher density, yokukansan cotreatment decreased the binding affinity (increased *K*_*d*_) of the 5-HT_1A_ receptor in isolation-stressed mice. Allosteric regulation is a function that regulates the binding affinity of the receptor to a ligand, and it has been reported that the 5-HT_1A_ receptor is allosterically modulated by Zn^2+^ [26]. Also, the adenosine A2A receptor [27], *μ*-opioid receptor [28], 5-HT_7_ receptor [29, 30], and FGFR_1_ receptor [31] can form heterodimers with the 5-HT_1A_ receptor and alter signaling. Determining the effects of yokukansan on these processes may help explain the underlying decrease in 5-HT_1A_ receptor-binding affinity.

We then examined functional alteration of the 5-HT_1A_ receptor in yokukansan cotreated isolation-stressed mice. The function of 5-HT_1A_ receptor in individual animals can be evaluated by differences in behavior after administration of a 5-HT_1A_ receptor agonist. Such responses include anxiolytic-like behavior in the open field and/or elevated plus maze [22, 32, 33], 5-HT syndrome (flat body posture, Straub tail response, hind limb abduction, forepaw treading, and head weaving behavior) [34–37], and hypothermic responses [38, 39]. A dose-dependent decrease in rearing was observed following administration of buspirone, a 5-HT_1A_ receptor partial agonist, in the open field or elevated plus maze [32, 33]. Furthermore, it has been reported that mice overexpressing 5-HT_1A_ receptors specifically in the cerebral cortex and dentate gyrus show an augmented dose-dependent decrease in open field rearing following administration of the 5-HT_1A_ receptors full agonist 8-OH-DPAT compared with wild-type mice (i.e., greater 8-OH-DPAT sensitivity; [22]). This decrease in rearing was thought to be mediated by postsynaptic 5-HT_1A_ receptors because these transgenic mice did not exhibit changes in brain 5-HT and 5-hydroxyindoleacetic acid levels or 5-HT turnover, which are decreased by stimulation of presynaptic 5-HT_1A_ autoreceptors in the raphe nuclei [40–42]. Thus, the change in rearing behavior in response to 8-OH-DPAT was considered to reflect the function of postsynaptic 5-HT_1A_ receptors in the cerebral cortex (including the PFC) and the dentate gyrus [22].

We therefore analyzed the responsiveness to 8-OH-DPAT by testing rearing behavior in the open field as an indicator of 5-HT_1A_ receptor function. Rearing behavior was not decreased by 0.3 mg/kg 8-OH-DPAT in group-housed mice but was decreased by this same dose in isolation-stressed mice. Moreover, yokukansan cotreatment reinforced this decreased response. This decrease in rearing was unlikely to be due to a general reduction in motor activity since the total distance traveled did not decline following 8-OH-DPAT. Based on these results, we conclude that administration of yokukansan enhanced the 5-HT_1A_ receptor-dependent reduction in rearing behavior in socially isolated mice.

Rearing behavior generally reflects the exploratory behavior of animals but may be an indicator of anxiety when the animal is placed in a novel environment (e.g., open field), with decreased rearing behavior associated with lower anxiety [42]. Indeed, repeated administration of buspirone, an anxiolytic psychotropic drug, decreased rearing behavior in the elevated plus maze [33]. Therefore, augmentation of the 8-OH-DPAT-induced decrease in rearing in yokukansan-treated mice suggests that repeated yokukansan administration reinforces the 5-HT_1A_ receptor-mediated anxiolytic effect.

Under the stressful condition of social isolation, repeated yokukansan administration increased the number of 5-HT_1A_ receptors in the PFC and enhanced the function of this receptor subtype. While the relation between these two effects remains unclear, it appears that repeated yokukansan administration alters brain function, and this may be the reason that repeated administration is required for this Kampo medicine to exert its pharmacological effects. Also, in our preliminary study, yokukansan did not increase the number of 5-HT_1A_ receptors in the PFC of group-housed mice (data not shown). Thus, the increasing effect of yokukansan on the number of 5-HT_1A_ receptors is thought to be predominantly exerted under stressful conditions.

GM found in Uncaria Hook, an active component of yokukansan, alleviates aggressive behavior in socially isolated mice through a partial agonist effect on the 5-HT_1A_ receptor [19]. A recent basic study proposed that GM could be absorbed into the blood after the oral administration of yokukansan and then reach the brain by crossing the blood-brain barrier [43]. More recently, we showed that GM was specifically bound in the frontal cortical region including the PFC, in which GM recognized 5-HT_1A_ receptors [44]. Thus, the present finding that repeated yokukansan administration increased the number of 5-HT_1A_ receptors and enhanced their response leads us to hypothesize that repeated yokukansan administration causes a functional change in 5-HT_1A_ receptors in the PFC to enhance the psychotropic effects of GM. Future studies are needed to investigate the active ingredients and mechanisms underlying the action of yokukansan on 5-HT_1A_ receptors.

## 5. Conclusion

The present results suggest that repeated administration of yokukansan increases 5-HT_1A_ receptor in the PFC of socially isolated mice, quantitatively and functionally. These mechanisms may explain the need for repeated administration of this Kampo medicine to ameliorate anxiety and aggression.

## Abbreviations

5-HT: Serotonin
5-HT_1A_: Serotonin 1A
8-OH-DPAT: 8-Hydroxy-2-(di-n-propylamino)tetralin
AD: Alzheimer's disease
ANOVA: Analysis of variance
BPSD: Behavioral and psychological symptoms of dementia
EDTA: Ethylenediaminetetraacetic acid
EGTA: Ethylene glycol tetraacetic acid
GAPDH: Glyceraldehyde-3-phosphate dehydrogenase
GM: Geissoschizine methyl ether
PCR: Polymerase chain reaction
PFC: Prefrontal cortex
PVDF: Polyvinylidene difluoride
YKS: Yokukansan.
